# Performance of ChatGPT-4o on the Japanese Medical Licensing Examination: Evalution of Accuracy in Text-Only and Image-Based Questions

**DOI:** 10.2196/63129

**Published:** 2024-12-24

**Authors:** Yuki Miyazaki, Masahiro Hata, Hisaki Omori, Atsuya Hirashima, Yuta Nakagawa, Mitsuhiro Eto, Shun Takahashi, Manabu Ikeda

**Affiliations:** 1 Department of Psychiatry, Osaka University Graduate School of Medicine, Suita, Japan; 2 Department of Psychiatry, Shichiyama Hospital, Sennan District, Japan; 3 Department of Psychiatry, Osaka Psychiatric Medical Center, Hirakata, Japan; 4 Department of Psychiatry, Asakayama General Hospital, Sakai, Japan; 5 Clinical Research and Education Center, Asakayama General Hospital, Sakai, Japan; 6 Graduate School of Rehabilitation Science, Osaka Metropolitan University, Habikino, Japan; 7 Department of Neuropsychiatry, Wakayama Medical University, Wakayama, Japan

**Keywords:** medical education, artificial intelligence, clinical decision-making, GPT-4o, medical licensing examination, Japan, images, accuracy, AI technology, application, decision-making, image-based, reliability, ChatGPT

## Abstract

This study evaluated the performance of ChatGPT with GPT-4 Omni (GPT-4o) on the 118th Japanese Medical Licensing Examination. The study focused on both text-only and image-based questions. The model demonstrated a high level of accuracy overall, with no significant difference in performance between text-only and image-based questions. Common errors included clinical judgment mistakes and prioritization issues, underscoring the need for further improvement in the integration of artificial intelligence into medical education and practice.

## Introduction

Artificial intelligence (AI) models, like ChatGPT [1], have shown promise in answering medical questions and assisting in clinical decision-making. Previous studies have evaluated AI performance on medical exams such as the United States Medical Licensing Examination (USMLE), where ChatGPT (GPT-3) achieved correct response rates of 42%‐64% on step 1 and 2 exams [2]. Studies on the Japanese Medical Licensing Examination (JMLE) reported that GPT-4 achieved 77.7% correct responses on 292 questions in 2022 (the 116th JMLE) [3] and 79.9% on 254 questions in 2023 (the 117th JMLE) [4]. GPT-4, using prompt tuning, achieved 82.7% on essential questions and 77.2% on basic and clinical questions among 336 questions [5]. GPT-4 Vision scored 78.2% on 386 questions, with significantly lower performance on image-based (71.9%) and table-based questions (35%) [6]. No studies have evaluated an AI model on all 400 JMLE questions. ChatGPT with GPT-4 Omni (GPT-4o), released May 13, 2024, represents significantly more natural human-computer interaction; it can accept input as text, audio, images, and video and create output as text, audio, and images [7], promising improved performance on image-based questions. Recent research has shown that GPT-4 has superior performance on psychiatric licensing examinations, emphasizing its potential in various medical fields [8]. As generative AI is increasingly applied in medical education, understanding its limitations will be essential for effectively integrating it into learning and practice. This study aimed to evaluate the performance of ChatGPT-4o on the JMLE, specifically assessing its ability to handle both text- and image-based questions. We hypothesized that ChatGPT-4o would demonstrate high proficiency in answering these questions, potentially meeting the JMLE passing criteria.

## Methods

### Overview

ChatGPT-4o was used from May 13 to May 19, 2024, to complete all 400 questions of the 118th JMLE, which was held in February 2024 [9]. The model, updated with data up to May 2023, was assessed on both text-only and image-based questions. The Japanese-language questions and multiple-choice responses were input verbatim without prompt engineering or memory functions. Images were also input when present.

### Statistical Analysis

To compare the correct response rates between the image-based and text-only questions, an independent sample, 2-tailed *t*-test was used. Statistical significance was set at *P*<.05 for all 2-tailed tests. All statistical analyses used Python’s *SciPy* library (v1.13.1).

### Ethical Considerations

This study used previously available data and no human participants. Therefore, ethics approval was not mandated.

## Results

### Evaluation Outcomes

Accuracy overall was 93.25%, with 93.48% for image-based questions and 93.18% for text-only questions (Table 1).

**Table 1.** Performance comparison of ChatGPT with GPT-4 Omni across different sections in the 118th Japanese Medical Licensing Examination.

Characteristics	Correct responses among all questions, n/N (%)	Correct responses among text-only questions, n/N (%)	Correct responses among image-based questions, n/N (%)
Overall	373/400 (93.2)	287/308 (93.2)	86/92 (93.5)
Section A (A001-A075)	71/75 (94.7)	42/43 (97.7)	29/32 (90.6)
Section B (B001-B050)	46/50 (92)	39/43 (90.7)	7/7 (100)
Section C (C001-C075)	68/75 (90.7)	61/68 (89.7)	7/7 (100)
Section D (D001-D075)	71/75 (94.7)	43/45 (95.6)	28/30 (93.3)
Section E (E001-E050)	48/50 (96)	46/48 (95.8)	2/2 (100)
Section F (F001-F075)	69/75 (92)	56/61 (91.8)	13/14 (92.9)

The correct response rate was not significantly different for text-only and image-based questions (*t*_5_=−1.190; *P*=.26).

### Error Classification

Errors made by ChatGPT-4o were analyzed and classified into 4 categories: diagnostic, logical, medical knowledge, and clinical judgment (Table 2). This classification system was developed and applied by multiple researchers with medical backgrounds; discrepancies were resolved through discussion.

**Table 2.** Classification and details of all errors of ChatGPT with GPT-4 Omni in the 118th Japanese Medical Licensing Examination.

Problem number	Classification	Error details
A021	Diagnostic error	Incorrect diagnosis: ChatGPT acknowledged multiple diagnostic possibilities but ultimately selected an incorrect option
A039	Logical error	Incorrect logic regarding risk reduction for blister package ingestion
A059	Medical knowledge error	Incorrect use of medical knowledge regarding the urea breath test
A061	Logical error	Incorrect final answer despite correct assessment of individual questions
B021	Medical knowledge error	Incorrect medical knowledge regarding the risk relationship of latex allergy after banana ingestion
B038	Medical knowledge error	Incorrect medical knowledge for classifying activity restriction
B047	Medical knowledge error	Incorrect medical knowledge about social support systems
B049	Medical knowledge error	Incorrect medical knowledge for describing the Trousseau sign
C012	Logical error	Correct medical knowledge but incorrect final answer (confusion between right and left)
C020	Medical knowledge error	Incorrect medical knowledge regarding occupational cataract risk
C040	Clinical judgment error	Incorrect triage decision, suggesting a black tag for a critically ill patient
C043	Clinical judgment error	Incorrect clinical judgment, prioritizing ultrasound over cardiotocogram
C055	Medical knowledge error	Incorrect medical knowledge regarding fetal rotation
C056	Logical error	Incorrect interpretation of the problem statement
C074	Medical knowledge error	In a case of hyperosmolar hyperglycemic syndrome, recommendation of a hypotonic solution instead of the correct choice of normal saline (0.9% sodium chloride)
D012	Medical knowledge error	Incorrect medical knowledge regarding chronic kidney disease severity classification
D017	Diagnostic error	Incorrect diagnosis: failure to accurately integrate textual and image data, leading to an erroneous diagnostic conclusion
D035	Medical knowledge error	In a case of metabolic alkalosis, failure to consider the importance of lactate-free solution
D047	Diagnostic error	Incorrect diagnosis: selection of the wrong option without considering or mentioning other differential diagnoses
E034	Medical knowledge error	Incorrect medical knowledge regarding postprandial blood glucose targets in gestational diabetes management
E041	Medical knowledge error	Incorrect medical knowledge for Glasgow Coma Scale motor response
F001	Medical knowledge error	Incorrect medical knowledge regarding the design principles of tactile paving
F010	Medical knowledge error	Incorrect medical knowledge regarding the peak population year in Japan
F018	Medical knowledge error	Correct image interpretation but incorrect medical knowledge regarding sagittal suture alignment
F054	Clinical judgment error	Incorrect decision on referring to a specialized hospital versus a community support hospital
F066	Logical error	Incorrect interpretation and judgment regarding wheelchair options
F068	Logical error	Incorrect interpretation of the problem statement regarding creatinine clearance calculation

## Discussion

ChatGPT-4o achieved an overall correct response rate of 93.2% on the 2024 (118th) JMLE without prompt engineering or memory functions, surpassing prior GPT models. Its performance did not decline on image-based or table-based questions, marking a significant improvement in multimodal question handling. This suggests that integrating multimodal capabilities may have significantly enhanced its clinical decision-making skills.

ChatGPT-4o’s performance meets the 118th JMLE passing criteria [10], which require (1) at least 160/200 points for compulsory questions (sections B and F); (2) at least 230/300 points for noncompulsory questions (sections A, C, D, and E); and (3) no more than 3 incorrect choices in contraindicated options, which remain undisclosed.

Although ChatGPT-4o met criteria (1) and (2), some responses suggest problematic clinical judgment. In question C040, the model incorrectly suggested a black tag (deceased/expectant) for a critically ill patient during triage, when the correct answer was a red tag (an immediate life-threatening condition). This error could have severe consequences in real-world emergency situations, potentially denying urgent care to a rescuable patient. In question C043, it incorrectly prioritized ultrasound over cardiotocography in a clinical decision. These errors highlight the potential for AI models to make clinical errors in judgment, as GPT-4o struggled with questions requiring clinical prioritization. This critical skill will become increasingly important in medical education.

These findings underscore the need for continued enhancement of AI models to ensure reliable and accurate clinical decision-making. Understanding and addressing these limitations will be critical for effectively integrating AI into medical education and practice.

## Abbreviations

AI: artificial intelligence
GPT-4o: GPT-4 Omni
JMLE: Japanese Medical Licensing Examination
USMLE: United States Medical Licensing Examination
